# Influence of Different Coupling Modes on the Robustness of Smart Grid under Targeted Attack

**DOI:** 10.3390/s18061699

**Published:** 2018-05-24

**Authors:** WenJie Kang, Gang Hu, PeiDong Zhu, Qiang Liu, Zhi Hang, Xin Liu

**Affiliations:** 1 College of Computer, National University of Defense Technology, Changsha 410073, China; kangwenjie@nudt.edu.cn (W.K.); hugang@nudt.edu.cn (G.H.); qiangliu06@nudt.edu.cn (Q.L.); 2 Department of Electronic Information and Electrical Engineering, Changsha University, Changsha 410022, China; pdzhu@nudt.edu.cn; 3 Key Laboratory of Hunan Province for Mobile Business Intelligence, Changsha 410205, China; hangzhi75925@163.com; 4 Department of Computer Engineering & Applied Math, Changsha University, Changsha 410022, China

**Keywords:** load redistribution, local coupling in subnets, node importance, assortative coupling in subnets, disassortative coupling in subnets, random coupling in subnets, the top-down coupling link, the bottom-up coupling link

## Abstract

Many previous works only focused on the cascading failure of global coupling of one-to-one structures in interdependent networks, but the local coupling of dual coupling structures has rarely been studied due to its complex structure. This will result in a serious consequence that many conclusions of the one-to-one structure may be incorrect in the dual coupling network and do not apply to the smart grid. Therefore, it is very necessary to subdivide the dual coupling link into a top-down coupling link and a bottom-up coupling link in order to study their influence on network robustness by combining with different coupling modes. Additionally, the power flow of the power grid can cause the load of a failed node to be allocated to its neighboring nodes and trigger a new round of load distribution when the load of these nodes exceeds their capacity. This means that the robustness of smart grids may be affected by four factors, i.e., load redistribution, local coupling, dual coupling link and coupling mode; however, the research on the influence of those factors on the network robustness is missing. In this paper, firstly, we construct the smart grid as a two-layer network with a dual coupling link and divide the power grid and communication network into many subnets based on the geographical location of their nodes. Secondly, we define node importance (NI) as an evaluation index to access the impact of nodes on the cyber or physical network and propose three types of coupling modes based on NI of nodes in the cyber and physical subnets, i.e., Assortative Coupling in Subnets (ACIS), Disassortative Coupling in Subnets (DCIS), and Random Coupling in Subnets (RCIS). Thirdly, a cascading failure model is proposed for studying the effect of local coupling of dual coupling link in combination with ACIS, DCIS, and RCIS on the robustness of the smart grid against a targeted attack, and the survival rate of functional nodes is used to assess the robustness of the smart grid. Finally, we use the IEEE 118-Bus System and the Italian High-Voltage Electrical Transmission Network to verify our model and obtain the same conclusions: (I) DCIS applied to the top-down coupling link is better able to enhance the robustness of the smart grid against a targeted attack than RCIS or ACIS, (II) ACIS applied to a bottom-up coupling link is better able to enhance the robustness of the smart grid against a targeted attack than RCIS or DCIS, and (III) the robustness of the smart grid can be improved by increasing the tolerance α. This paper provides some guidelines for slowing down the speed of the cascading failures in the design of architecture and optimization of interdependent networks, such as a top-down link with DCIS, a bottom-up link with ACIS, and an increased tolerance α.

## 1. Introduction

As an application scenario of the Internet of Things (IoT), smart grids are developing rapidly with a structure based on a two-layer network with dual coupling link. They are also facing many challenges that attract a large number of researchers for increasing the profit attained, enhancing system reliability, reducing electricity cost, improving the robustness, reducing the risk of being attacked, and developing defense mechanisms against different attack strategies. From the perspective of energy management optimization and energy efficiency improvements, Marzband et al. [1] proposed improved versions of the popular optimization techniques that include particle swarm optimization (PSO), harmony search (HS), differential evolution (DE) and the bat algorithm (BAT) to solve the non-linear and non-convex Market Operator Transactive Energy (MO-TE) structure problem. From the perspective of network security, He et al. [2] provided a comprehensive and systematic review of the critical cyber-physical attack threats and defense strategies in the smart grid. Liu et al. [3] discussed security threats and defensive techniques of machine learning from a data driven perspective. However, this paper will study the effect of different coupling modes applied to dual coupling links on the cascading failure of the smart grid from the perspective of improving network robustness.

Diverse critical infrastructures, usually represented as interdependent networks, are rarely isolated; rather, they are interdependent [4]. Most recently, research on complex networks was applied to interdependent networks by Buldyrev et al. [5]. This research revealed new perspectives and research approaches to explain the principle of cascading failures. However, previous works have mainly focused on network structure and have rarely considered real network load functions [6,7]. The dual coupling relationship and the load redistribution characteristic have a great influence on the cascading failures of interdependent networks. On the one hand, failed nodes may trigger load redistribution in the power grid, which can cause other nodes to overload and fail. On the other hand, the nodes in the communication network fail, which will cause more the coupled physical nodes in the power grid to fail. This, in turn, will result in the failure of the coupled cyber nodes in the communication network.

Buldyrev et al. [7,8] were the first to establish a framework for the analysis of catastrophic failures in interdependent networks [9]. This framework breaks through the frontier of complex networks theory that still focuses on a single, non-interactive network [10,11,12]. Inspired by this pioneering research, many works have used the critical size of the giant component to represent the functional integrity of the composite network [6,13,14,15]. Liu et al. [16] used the percolation framework to study the effect of weak node coupling strength across networks analytically and numerically on the robustness of interdependent networks and they found that there is a crossover point at which a first-order percolation transition changes to a second-order percolation transition. Kornbluth et al. [17] proposed the concept of the distance to study the effect of the proximity of interdependent nodes on the cascading failures against an initial attack and they found that there is a non-trivial relation between the degree of nodes and the maximum distance between coupled nodes. Buldyrev and co-workers proposed a perfect and complete theoretical system to analyze the percolation of different topologies of interdependent networks and laid a theoretical foundation for subsequent studies, which highlights the subtleties of this problem and clearly shows that systems made of interdependent networks, such as interdependent networks can be intrinsically more fragile than each isolated network [9].

From the perspective of functional properties, the load of nodes was taken into account when the authors established different models to study the cascading failure of interdependent networks in recent research literatures [18,19,20,21]. Many works have adopted degree [22,23], betweenness [24,25,26,27], or degree of degree [28] as the initial load of nodes. In addition, $w_{im}*w_{jn}$ was used as the initial load of an edge $e_{ij}$, where $w_{im}={\left(k_{i}*k_{m}\right)}^{\alpha }$ represents the coupled strength between two coupled nodes *i* and *m*, and $k_{i}$ is the degree of node *i* [19]. Similarly, $\lambda s_{i}^{\alpha }$ was used as the initial load of node *i*, where $s_{i}$ represents the total weights of all edges connected with node *i* [23]. When a node fails due to a targeted attack, the balance of the load is broken to cause load redistribution that may trigger more nodes to overload and fail. In the presence of over-load failure model, the studies presented a load-induced failure mathematical model to study the mechanism of the cascading failure of interdependent networks and explained why a few failed nodes can result in the breakdown of the entire network. These models provide us with an effective strategy to reduce the effect of load on the cascading failure of interdependent networks.

Wang et al. [29] focused on percolation-cascading process in BA-BA, ER-ER, and BA-ER coupled networks and proposed a stochastic structural algorithm to form coupling edges between two layers, and simulation shows that assortative network performs better in cascading failure process and BA network is more robust than other types of networks. Zhang et al. [30] used the memetic algorithm (MA) to optimize the coupling links of interdependent networks and compared MA optimized coupling strategy and traditional assortative, disassortative and random coupling preferences. They found that MA optimized coupling strategy with a moderate assortative value has an outstanding performance against cascading failures on both synthetic scale-free interdependent networks and real-world networks. Tan et al. [31] studied the influence of interconnections on traffic congestion in BA scale-free networks, and they found that assortative coupling is more helpful to ease traffic congestion than disassortative and random coupling when the node processing capacity is allocated according to node usage probability. The influence of different coupling preferences on the network robustness is investigated over interdependent networks based on a one-to-one structure. These works can be useful to the design and optimization of robust interdependent networks.

Recently, an increasing number of the details of interdependent networks have been considered, including coupling strength [15], support-dependence relations [32], coupling preferences [33], clustered structures [34], and community structure [22]. Chen et al. [35] studied the effect of coupling preference on systems’ robustness and used betweenness as node load that is used to connect nodes between layers in order to generate assortative, disassortative and random coupling links, and simulation shows that disassortative coupling is more robust for sparse coupling while assortative coupling performs better for dense coupling. Babaei et al. [36] found that the robustness of modular small-world networks is improved by increasing inter-community links in response to both random and targeted attacks. Tian et al. [22] found that increasing the inter-community connection can enhance the robustness of interdependent modular scale-free (SF) networks. Brummitt et al. [20] studied and estimated the effect of the optimal level of interconnectivity on the cascading failure of interdependent networks. They found that adding some connectivity between two isolated networks is beneficial in preventing the largest cascades in each system, while it becomes detrimental when the number of coupling link exceeds a certain value. The effect of different impact factors on the robustness of interdependent networks is investigated and many effective methods are provided for constructing a robust interdependent network.

The following deficiencies regarding research of the cascading failure in interdependent networks based on load redistribution have been highlighted in this paper:

- The limitation of the application scenario for the giant component. The concept of the giant component only applies to homogeneous networks, while it does not apply to heterogeneous networks. For instance, when the power grid is divided into several fragments by a targeted attack, the smaller components are still valid as long as the generation nodes and load nodes coexist therein.
- Lack of a cascading failure for considering dual coupling between the communication network and power grid. The one-to-one correspondence in the framework [37] cannot cover all the dependency situations in the real world and in most cases, for instance, the smart grid has dual coupling links [35] between the communication network and power grid.
- Lack of an algorithm to assess the importance of cyber or physical nodes according to node load and network characteristics. This does not reflect reality because the properties of network structure cannot represent the functional characteristics and cannot reflect the actual network situation.
- The unreasonableness of load definition. Network attributes (degree, betweenness, the degree of degree etc.) cannot be treated as node load in power grids because the load is related to voltage, active power, and reactive power.
- The limitation of increasing coupling strength. Increasing the coupling links will result in increased cost and reduced revenue, which is impractical and not the best choice.
- Lack of a model to analyze the effect of local coupling between two subnets on the robustness of smart grids. Global coupling increases the length of the coupling link, which increases costs and is impractical.

This paper is an extended version of the previous conference paper [38] and the main purpose of this study is to improve the robustness of interdependent networks by changing the coupling mode without increasing the coupling links. Since long-distance coupling links also increase costs, we divided the network into many small sub-networks based on the geographical distribution area of substations and used local coupling between cyber and physical subnets to study the robustness of interdependent networks. Local coupling only allows the nodes in cyber subnet $A_{1}$ to couple with nodes in physical subnet $B_{1}$, which has the same geographical area as $A_{1}$; therefore, it is crucial to study the influence of the local coupling in subnets on the robustness of the smart grid. In addition, the concept of “Giant Component” is not used in our cascading failure model in which only isolated nodes are considered invalid and smaller components are still functioning when generation nodes and load nodes coexist on the same component. As such, node survival rate is used to evaluate the robustness of the smart grid after a fraction 1−p of nodes is removed.

The contributions of this study can be summarized as follows:

- Dual coupling link is constructed into the framework of the smart grid, which contains the top-down coupling link and the bottom-up coupling link. Dual coupling network model reflects the real coupling relationship of the smart grid. Dual coupling relationship may have a great impact on cascading failure of the smart grid and may lead to completely different conclusions compared to the one-to-one coupling model.
- Load redistribution characteristic, network attributes, and coupling relationship are used to design an algorithm to assess the importance of nodes (NI). The nodes between physical and cyber subnets are connected based on NI to form Assortative Coupling in Subnets (ACIS), Disassortative Coupling in Subnets (DCIS), and Random Coupling in Subnets (RCIS).
- ACIS, DCIS, and RCIS are applied to the top-down coupling link and the bottom-up coupling link in order to study how to enhance the robustness of the smart grid.
- The voltage of nodes is used as its load and the impedance of link is used to calculate and allocate the load proportion of failed nodes to its neighboring nodes. The load redistribution algorithm is more in line with the actual situation of the power grid.
- The effect of local coupling between two subnets on the robustness of the smart grid is considered. The communication network and power grid can be divided into multiple subnets according to the geographical distribution of nodes. The local coupling can reduce the length of coupling links and reduce costs.

This paper is organized as follows. In Section 2, we introduce related research on the cascading failures of interdependent networks. In Section 3, we propose three different coupling modes in order to study their effect on the robustness of the smart grid. The cascading failure model is described, and the survival rate of functional nodes is used as an evaluation index for assessing the robustness of the smart grid in Section 4. Section 5 gives two case studies, two datasets, i.e., the IEEE 118-Bus System and the Italian High-Voltage Electrical Transmission Network. Two experimental results draw the same conclusion that top-down coupling links with DCIS and bottom-up coupling links with ACIS are more beneficial in enhancing the robustness of the smart grid than those with other coupling modes. Section 6 summarizes the relevant conclusions and presents suggestions for future research in this area.

## 2. Related Work

Gao et al. [39] proposed a framework for studying the percolation of *n* interdependent networks. Zhou et al. [40] found that the internal node correlations in each of the two interdependent networks significantly change the critical density of failures and that the assortativity within a single network decreases the robustness of the entire system. Han et al. [23] proposed a load-capacity model for analyzing the cascading failure in both interdependent and isolated networks, and they found that network robustness is positively related to the capacity and is negatively related to the load. Qiu et al. [41] studied the optimal weighting scheme and the role of coupling strength against load failures in symmetrically and asymmetrically coupled interdependent networks. They found that the symmetrically and asymmetrically coupled interdependent networks achieve robustness and better cost configuration against overload-induced failure, in which case coupling strength was found to be weaker. Qiu et al. [42] studied load cascading dynamics in a system composed of coupled interdependent networks while adopting a local weighted flow redistribution rule, and they found that increasing the intra- or inter-connectivity is beneficial in enhancing the robustness of interdependent networks. Liu et al. [16] used the percolation framework to study the effect of coupling strength of nodes on the robustness of interdependent networks.

Shao et al. [32] studied the cascading failures in two coupled networks, wherein multiple support–dependence relations are randomly built. Parshani et al. [15] studied a system composed of two interdependent networks and found that reducing the coupling strength leads to a change from a first-order percolation phase transition to a second-order percolation transition at a critical point. Huang et al. [34] developed an analytical method for studying how clustering within the single network of interdependent networks affects its robustness, and they found that clustering significantly increases the vulnerability of interdependent networks. Tan et al. [27] proposed a global load redistribution model to study the cascading failure in interconnected networks. They found that the sparsely interconnected networks are fragile while densely interconnected ones are robust. They also discovered that the interconnected networks using assortative coupling are more robust than those that use the disassortative or random coupling. Tian et al. [43] investigated two clustered networks with both interdependent and interconnected links. They found that clustering significantly changes the robustness of networks with strong dependency coupling strength. Dong et al. [44] analyzed the percolation behaviors of clustered networks with partial support-dependence relations and found that the clustering coefficient has a significant impact on the robustness of interdependent networks in the case of strong coupling strength, but that it has little influence in the case of weak coupling strength.

Cheng et.al. [45] developed a theoretical framework for studying the robustness of interdependent networks coupled with different type networks under both targeted and random attacks. Zhang et al. [25] analyzed the effect of network size on the robustness of interconnected networks under a targeted attack. They found that the larger sized network is more robust for sparse coupling, while it is more fragile for dense coupling. Shao et al. [46] applied a study on the clustering of two fully coupled networks and applied it to partially interdependent networks with clustering. Tian et al. [22] investigated cascading failures in interdependent modular scale-free networks under inner attacks and hub attacks from the global and local perspectives. They found that the assortative coupling in communities (ACIC) is more beneficial in resisting cascading failures than random coupling in communities (RCIC) and assortative coupling with communities (ACWC). Chen et al. [18] studied the cascading failure of interdependent networks with different coupling preferences under a targeted attack. They found that disassortative coupling is more robust than assortative coupling for sparse coupling while assortative coupling performs better for dense coupling than disassortative coupling. Wang et al. [33] studied the effect of different coupling preferences on the cascading failure of interdependent networks. They found that an assortative coupling network has a smaller proportion of the largest connected subgraph than other coupling networks and that the failure speed of the iteration step of an assortative coupling network is slower than other coupling networks.

## 3. The Coupling Model of the Smart Grid

**Figure 1:**
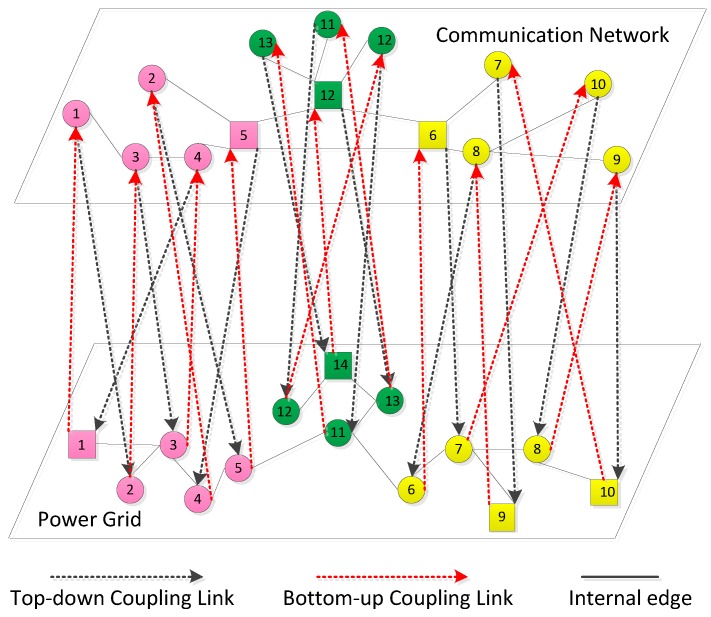
The framework of a smart grid divided into a communication network and a power grid. Different colored nodes form different subnets. The top-down coupling link is coupling edge from a cyber node to a physical node. The bottom-up coupling link is coupling edge form a physical node to a cyber node.

A smart grid is a two-layer network that is coupled by a power grid and a communication network. Figure 1 shows two-layer network structure and dual-local coupling mode of a smart grid. The upper layer is the communication network where the square node represents the control center and the circular nodes represent sensors. The lower layer is the power grid where square nodes represent generators and the circular nodes represent substations. Each layer can be divided into many subnets in terms of geographical factors and each subnet can be treated as an autonomous system that is represented by the same color network in Figure 1. The edge of interdependent networks is divided into two types: internal edge and coupling edge. The internal edge connects any two nodes in a single-layer network and is shown by solid lines in Figure 1. The coupling edge contains the top-down coupling link (C→P) and the bottom-up coupling link (P→C). *P* and *C* represent the physical layer and the cyber layer, respectively. C→P represents that the physical nodes depend on the cyber nodes, which is shown by black dotted lines with arrows in Figure 1. P→C represents that the cyber nodes depend on the physical nodes, which is shown as red dotted lines with arrows in Figure 1.

**Definition** **1.** The smart grid is defined as SG={V,E,R}, where the node set $V=\{V^{P},V^{C}\}$ contains node set $V^{P}$ of a power grid and the node set $V^{C}$ of a communication network. $E=\{E^{P},E^{C}\}$ represents internal edge, which contains edge set $E^{P}$ of the power grid and the edge set $E^{C}$ of the communication network. $R=\{r_{ij}|i\in \;V^{P},j\in V^{C}\;or\;i\in V^{C},j\in V^{P}\}$ represents the coupling relationship matrix, which contains the top-down and bottom-up coupling links. In the power grid, $V^{P}=\{v_{1}^{G},v_{2}^{G},$…$,v_{m}^{G},v_{1}^{L},v_{2}^{L},$…$,v_{n}^{L}\}$ represents the physical node set, where $v_{i}^{G}$ represents generation node i and $v_{j}^{L}$ represents load node j, which contains transmission nodes and distribution nodes. In the communication network, $V^{C}=\{v_{1}^{C},v_{2}^{C},$…$,v_{k}^{C},v_{1}^{S},v_{2}^{S},$…$,v_{l}^{S}\}$ represents the cyber node set, where $v_{i}^{C}$ represents the control center node i and $v_{j}^{S}$ represents sensor node j.

The coupling relationship matrix *R* is used to describe the dependencies between nodes in the power grid and the communication network. Formula (1) represents a bottom-up coupling relationship matrix from the physical nodes to the cyber nodes, while Formula (2) represents a top-down coupling relationship matrix from the cyber nodes to the physical nodes. $R_{PC}\left(i,j\right)=r_{p_{i}\to c_{j}}=1$ indicates that the node *j* in the communication network depends on the node *i* in the power grid. $R_{CP}\left(j,i\right)=r_{c_{j}\to pi}=1$ indicates that node *i* in the power grid depends on the node *j* in the communication network. $R_{PC}\left(i,j\right)$ or $R_{CP}\left(j,i\right)=0$ indicates that there is no dependence. Here, special explanation $\left(R_{PC}\left(i,j\right)=1\right)\neq \left(R_{CP}\left(j,i\right)=1\right)$

(1) $$R_{PC}=\left[\begin{array}{cccc} r_{p_{1}\to c_{1}} & r_{p_{1}\to c_{2}} & \ldots & r_{p_{1}\to c_{n}}\\ r_{p_{2}\to c_{1}} & r_{p_{2}\to c_{2}} & \ldots & r_{p_{2}\to c_{n}}\\ \ldots & \ldots & \ldots & \ldots \\ r_{p_{n}\to c_{1}} & r_{p_{n}\to c_{2}} & \ldots & r_{p_{n}\to c_{n}} \end{array}\right]$$

(2) $$R_{CP}=\left[\begin{array}{cccc} r_{c_{1}\to p_{1}} & r_{c_{1}\to p_{2}} & \ldots & r_{c_{1}\to p_{n}}\\ r_{c_{2}\to p_{1}} & r_{c_{2}\to p_{2}} & \ldots & r_{c_{2}\to p_{n}}\\ \ldots & \ldots & \ldots & \ldots \\ r_{c_{n}\to p_{1}} & r_{c_{n}\to p_{2}} & \ldots & r_{c_{n}\to p_{n}} \end{array}\right]$$

### 3.1. The Node Importance of the Physical Nodes

A power grid is a heterogeneous network and has many functional properties, for instance, electric current, voltage, frequency, active power, and reactive power. The electric current flows from the generation nodes to the load nodes like water, which causes the phenomenon of the load of a failed node being redistributed to its neighbor nodes. The load used as the special feature of the power grid affects the function of the whole network. The total number of most efficient paths passing through node *i* is used as its initial load for establishing a model of cascading failure in the complex network.

Tian et al. [22] used the betweenness centrality as the initial load in order to study the influence of different coupling preferences on the cascading failure of modular scale-free networks. Similarly, the number of the shortest paths between pairs of nodes over the network passing through the node *i* has been used as the initial load in [47,48]. Yan et al. [28] utilized the degree of degree as the initial load for analyzing multi-contingency cascading of smart grid based on a self-organizing map. Wang et al. [19] defined the coupled strength between two coupled nodes as the initial load of an edge in order to study the cascading failure of interdependent networks. Hen et al. [23] used the total weights of all edges connected with node *i* as its initial load to simulate load-induced cascading failure in asymmetrical interdependent networks.

However, the above literatures all feature a certain irrationality in using network structure attributes (e.g., degree, betweenness, the degree of degree, and coupled strength etc.) as functional attributes (e.g., the load, plow flow, data flow, voltage, frequency etc.). A sufficiently sophisticated attack could result in potentially hazardous below or above the voltage on a power node, which may destroy consumer equipment [49]. In the actual situation, the voltage which is too high or low may damage the transformer or trigger the automatic tripping of the transformer to cause a large-scale blackout; therefore, the voltage is considered as the load of a node. The initial load of node *i* is described as:

(3) $$L\left(v_{i}\right)=Vol_{i}$$

**Definition** **2.** *The capacity of the node is defined as a kind of tolerance ability to withstand load changes, which indicates that the power system can still operate normally after the load has increased or decreased within a certain range. The capacity of node i can be expressed as*

(4) $$C\left(v_{i}\right)=\left(1\pm \alpha \right)*L\left(v_{i}\right)$$

*where α represents the tolerance parameter and ± indicates the capacity of nodes that can withstand the range of voltage variation rate. If the voltage variation rate of a node is over α or below α, it will fail. This means that a high voltage or low voltage outside of the tolerance range can lead to the failure of a node.*

Function $\Delta f_{ij}$ denotes the proportion of load distribution from node *i* to node *j*. The load of a failed node is distributed to the adjacent nodes by computing the impedance of the link between two nodes. The new added load $\Delta L(x_{j})$ depends on the initial load and the proportion of load distribution $\Delta f_{ij}$. If the sum of initial load of node *j* and the partial load from node *i* exceeds the capacity of node *j*, node *j* fails. This leads to a new round of load redistribution. This process repeats until there is no overloaded node or the entire network is paralyzed.

(5) $$\Delta f_{ij}=\beta *\frac{1+(I_{Max}-I_{ij})}{\left(\sum_{k\in B(i)}I_{ik}\right)}$$

(6) $$\Delta L\left(v_{j}\right)=\left(1+\Delta f_{ij}\right)*L\left(v_{i}\right)=\left(1+\beta *\frac{1+(I_{Max}-I_{ij})}{\left(\sum_{k\in B(i)}I_{ik}\right)}\right)*L\left(v_{i}\right)$$

where B(i) denotes the neighboring nodes set of node *i*, $I_{ij}$ denotes the impedance of the branch between node *i* and *j*, β is a parameter that determines the increase or decrease of the neighboring nodes and β=1 denotes that the change $\Delta f_{ij}$ of node *i* is added to neighboring node *j*, β=−1 denotes that the load of neighboring node j reduces the rate $\Delta f_{ij}$ due to the lack of power-supply for node *i*. $(\frac{1+(I_{Max}-I_{ij})}{\left(\sum_{k\in B(i)}I_{ik}\right)})$ indicates that the impedance of the link $e_{ij}$ has an impediment to the power flow passing through it. The link $e_{ij}$ with a greater impedance will be passed by a smaller proportion of the power flow, which means that the load distributed by node *i* to its neighboring nodes *j* is also small.

**Definition** **3.** *Node importance $NI_{i}^{P}$ is used as a significant evaluation index to assess the impact of nodes on the power grid; where $f_{i}^{P}$ represents the failure node set in which all nodes become invalid after a node i is removed, $n(f_{i}^{P})$ denotes the number of failed nodes, $R_{PC}\left(i,j\right)=1$ denotes that there is a coupling link from a physical node i to a cyber node j, and $DoD_{i}$ is the degree of degree of node i, which represents the sum of the degree of its neighboring nodes. $DoD_{Max}$ is the maximum value of all degree of degrees (DoDs). NI is written as:*

(7) $$NI_{i}^{P}=n\left(f_{i}^{p}\right)+\sum_{R_{PC}\left(i,j\right)=1}\frac{DoD_{j}}{DoD_{Max}}$$

The size $n(f_{i}^{p})$ of FNS of node *i* can be obtained by calculating Algorithm 1 that can be expressed as follows.

**Step 1: (Initialization)** Obtain the information of all nodes (e.g., physical node set $V^{P}$, load *L*, tolerance α) and the impedance $I_{ij}$ of all branches.

**Step 2: (Node Failure)** Remove a node from the physical node set $V^{P}$ and add it to failure node set (FNS).

**Step 3: (Load Redistribution)** If the removed node is a load node, the load is distributed to the neighboring node by applying Formula (6) and β=1. If the removed node is a generation node, the load of its neighboring nodes changes to zero instantly. Then, the load of its neighboring node *j* reduces the rate $\Delta L(v_{j})$ due to the lack of power-supply of node *i* by applying Formula (6) and β=−1.

**Step 4: (Judgment of failed nodes)** If the load of a node exceeds the range of its capacity, it is considered invalid and is added to FNS.

**Step 5: (Iteration)** A failed neighboring node will trigger a new round of load redistribution and **steps 2**–**4** are repeated until there is no overloaded node or the smart grid is paralyzed.

**Step 6: (Identifying** $n(f_{i}^{p})$**)** Computing the size of the FNS of the node. Repeat **steps 2**–**5** until all nodes are traversed.

**Algorithm 1** The Algorithm of Load Redistribution**Input:**
$V^{P}=\left\{v_{1}^{G},v_{2}^{G},\ldots ,v_{m}^{G},v_{1}^{L},v_{2}^{L},\ldots ,v_{n}^{L}\right\}$, α, *L*,$I_{ij}$ **Output:** Failure Node Set: fns  1:**function** getNumberofFailureNode($V^{P}$)  2: fns = null  3: $\Delta f_{ij}=\beta *\frac{1+(I_{Max}-I_{ij})}{\sum_{k\in B(i)}I_{ik}}$  4: **for** i=0; i<m+n−1;i++ **do**  5:  fns.add($V_{i}^{P}$)  6:  **if** $V_{i}^{P}\in G$ **then**  7:   $L\left(V_{j}^{P}\right)=L\left(V_{j}^{P}\right)(1-|\Delta f_{ij}\left|\right)$  8:  **end if**  9:  **if** $V_{i}^{P}\in L$ **then**10:   $L\left(V_{j}^{P}\right)=L\left(V_{j}^{P}\right)(1+|\Delta f_{ij}\left|\right)$11:  **end if**12: **end for**13: **if** $L\left(V_{j}^{p}\right)>C\left(V_{j}^{p}\right)\|L\left(V_{j}^{p}\right)<C\left(V_{j}^{p}\right)$ **then**14:  fns.add($V_{j}^{P}$)15:  **function** getNumberofFailureNode($V_{j}^{p}$)16:  **end function**17: **end if**18: **return** fns.size()19:**end function**

### 3.2. The Node Importance of the Cyber Nodes

The communication network is an abstract overview of the SCADA systems/ Energy Management Systems (EMS) in a smart grid. SCADA systems have been implemented to monitor and control electrical power grids for decades [50]. Industrial experience has shown that the practical deployment of SCADA based systems may be restricted to high-voltage transmission networks and is not suitable for the larger-scale monitoring and control of an entire electrical grid [51]. A distributed monitoring control system is named Information and Communication Technology (ICT) system, which is proposed to manage the power grid [52]. The communication network also contains many subnets, each of which has a control center and multiple sensors.

In fact, load redistribution also occurs in communication networks. When the data flow at a node exceeds its capacity, the node will refuse to provide service and will fail, and its data flow will be distributed to the neighboring nodes. If overload also occurs in these neighboring nodes, it will trigger a new round of load redistribution until there is no overloaded node or the entire network is paralyzed. As such, the node passed by the bigger data flow is considered an important node. However, we have no way to simulate such an experimental environment because the real-time features of data flow will bring uncertainty to the importance of cyber nodes. Therefore, we make reasonable assumptions as follows: (I) a node with a big degree also has a big data flow because its neighboring nodes need it to transmit data, and (II) isolated nodes are considered to be invalid, which may be caused by the failure of a large-degree node.

**Definition** **4.** *The (NI) of a cyber node depends on the degree of its nodes and the NI of its coupled physical nodes.*

(8) $$NI_{i}^{C}=k_{i}*\sum_{R_{CP}\left(i,j\right)=1}\frac{NI_{j}^{P}}{NI_{Max}^{P}}$$

*where $k_{i}$ is the degree of node i and $NI_{j}^{P}$ denotes the importance of the physical node j, $NI_{Max}^{P}$ is the maximum of NI of physical nodes, and $R_{CP}\left(i,j\right)=1$ denotes that there is a coupling link from a cyber node i to a physical node j. This means that the importance of the cyber node depends on its degree and the physical nodes that it controls.*

### 3.3. Three Coupling Modes Based on NI

There are three types of coupling modes: assortative coupling in subnets, disassortative coupling in subnets, and random coupling in subnets. There are two types of coupling edges: the top-down coupling link and the bottom-up coupling link. The top-down coupling link represents a control dependency that the cyber nodes provide the remote monitoring, measurement and controlling to the physical nodes. The bottom-up coupling link represents a power support independence, where the physical nodes provide power to the cyber nodes. We divide power grid *A* and communication network *B* into *N* subnets $A_{1}$, $A_{2}$, …, $A_{N}$ and $B_{1}$, $B_{2}$, …, $B_{N}$, respectively. We assume that networks with the same subscript are in the same geographical area, such as $A_{1}$ and $B_{1}$, $A_{2}$ and $B_{2}$, …, $A_{N}$ and $B_{N}$. Local coupling rules only allow nodes in $A_{1}$ to couple with nodes in $B_{1}$, similarly, nodes in $A_{2}$ to couple with nodes in $B_{2}$, and so on.

Random Coupling in Subnets (RCIS): A node in $A_{1}$ is randomly chosen to connect to a node in $B_{1}$ with one-to-one correspondence until all nodes are handled. This process is repeated until all subnets are handled.

Assortative Coupling in Subnets (ACIS): The subnets in the power grid and communication network are chosen by the same geographical area, respectively. The node with the largest NI in the selected subnet of the power grid is connected to the node with the largest NI in the communication network by one-to-one correspondence. The node with the second largest NI in the selected subnet of the power grid is connected to the node with the second largest NI in the communication network by one-to-one correspondence. This process is repeated until all nodes are handled. For instance, we sort nodes in $A_{1}$, $A_{2}$, …, $A_{N}$ in descending order of NI, labeled as $a_{1}^{A_{1}}$, $a_{2}^{A_{1}}$, …, $a_{n}^{A_{1}}$, $a_{1}^{A_{2}}$, $a_{2}^{A_{2}}$, …, $a_{m}^{A_{2}}$, …, $a_{1}^{A_{N}}$, $a_{2}^{A_{N}}$, …, $a_{k}^{A_{N}}$. The nodes in $B_{1}$, $B_{2}$, …, $B_{N}$ are sorted in the same way, labeled as $b_{1}^{B_{1}}$, $b_{2}^{B_{1}}$, …, $b_{n}^{B_{1}}$, $b_{1}^{B_{2}}$, $b_{2}^{B_{2}}$, …, $b_{m}^{B_{2}}$, …, $b_{1}^{B_{N}}$, $b_{2}^{B_{N}}$, …, $b_{k}^{B_{N}}$. Then, connections are made between $a_{1}^{A_{1}}$ and $b_{1}^{B_{1}}$, $a_{2}^{A_{1}}$ and $b_{2}^{B_{1}}$, and so on. This process is repeated until all interconnected links are added between *A* and *B*.

Disassortative Coupling in Subnets (DCIS): The subnets in the power grid and communication network are chosen by the same geographical area. Then, the node with the largest NI in the selected subnet of the power grid is connected to the node with the smallest NI in the communication network by one-to-one correspondence. The node with the second largest NI in the selected subnet of the power grid is connected to the node with the second smallest NI in the information network by one-to-one correspondence. This process is repeated until all nodes are handled. For instance, we sort nodes in $A_{1}$, $A_{2}$, …, $A_{N}$ in descending order of NI, labeled as $a_{1}^{A_{1}}$, $a_{2}^{A_{1}}$, …, $a_{n}^{A_{1}}$, $a_{1}^{A_{2}}$, $a_{2}^{A_{2}}$, …, $a_{m}^{A_{2}}$, $a_{1}^{A_{N}}$, $a_{2}^{A_{N}}$, …, $a_{k}^{A_{N}}$. The nodes in $B_{1}$, $B_{2}$, …, $B_{N}$ are sorted in ascending order of NI, labeled as $b_{1}^{B_{1}}$, $b_{2}^{B_{1}}$, …, $b_{n}^{B_{1}}$, $b_{1}^{B_{2}}$, $b_{2}^{B_{2}}$, …, $b_{m}^{B_{2}}$, $b_{1}^{B_{N}}$, $b_{2}^{B_{N}}$, …, $b_{k}^{B_{N}}$. Then, connections are made $a_{1}^{A_{1}}$ and $b_{1}^{B_{1}}$, $a_{2}^{A_{1}}$ and $b_{2}^{B_{1}}$, and so on. This process is repeated until all interconnected links are added between *A* and *B*.

## 4. Cascading Failure Model

**Figure 2:**
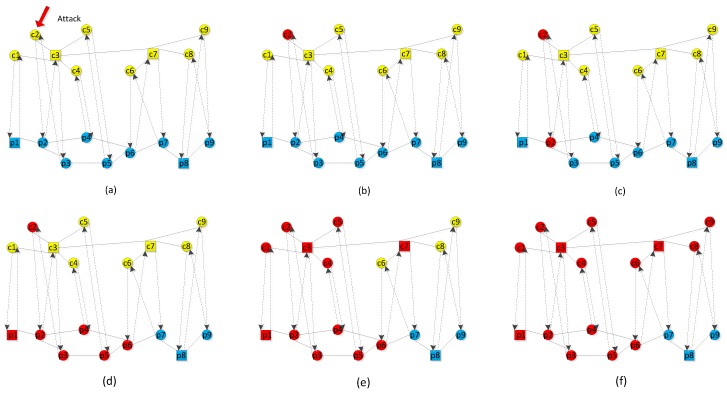
The cascading failure of the smart grid based on load redistribution. (**a**) The cyber node c2 is attacked. (**b**) c2 fails due to being attacked. (**c**) A failed c2 causes p2 to fail due to error control command. (**d**) A failed p2 triggers load redistribution and leads to the failure of p1, p3, p4, p5, and p6 due to overload. (**e**) c1, c3, c4, c5, and c7 fail due to lack of power supply from p1, p2, p4, p5, and p6. (**f**) c6, c8, and c9 fail due to becoming isolated nodes. The upper network is a communication network and its functioning nodes are marked in yellow. The lower network is a power grid and its functioning nodes are marked in blue. The failed nodes are marked in red.

An overload-induced failure takes place in the power grid, and different coupling modes may have different cascading failures. Figure 2 shows how an initial attack can damage an interdependent network due to overload-induced failure. The yellow nodes represent the cyber nodes c1, c2, …, c9, while the blue nodes represent the physical nodes p1, p2, …, p9. In the power grid, p1 and p8 are generators, while the other nodes are load nodes. In the communication network, c3 and c7 are the control centers, while the other nodes are sensors. The solid lines in the power grid and communication network represent the internal edges, while the dashed lines connecting the two networks represent the coupling edges. The link p1→c1 indicates that c1 depends on p1, while the link c1→p1 indicates that p1 is controlled by c1. Figure 2a shows that c2 has been attacked and fails. c2 is marked in red in Figure 2b. A failed c2 can cause p2 to fail due to error control commands, shown in Figure 2c. A failed p2 triggers load redistribution and the load of p2 is distributed to its neighbors. Because the load of nodes p1, p3 and p4 exceeds their capacity after having received some amount of load from p2, they fail due to overload. Similarly, failed p3 and p4 cause the failure of p5 and p6. However, p7, p8, and p9 are still active in Figure 2d. Nodes c1, c3, c4, c5 and c7 fail due to lack of power supply from p1, p2, p4, p5, and p6 in Figure 2e. Nodes c6, c8, and c9 fail due to becoming isolated nodes and the communication network breaks down in Figure 2f. This means that first-order phase transformation has happened in an interdependent network at this time and the smart grid has become a single network that is comprised of p7, p8, and p9.

**Definition** **5.** *The survival rate P of the functional nodes for assessing the robustness of an interdependent network is defined as the proportion of functional nodes in the smart grid after a fraction 1−p of nodes is removed and reflects the network robustness against a targeted attack. A smaller P indicates that cascading failure of interdependent networks has a faster diffusion rate and vice versa.*

(9) $$P=1-\frac{F^{P}+F^{C}}{N^{P}+N^{C}}$$

*where $N^{P}$ and $N^{C}$ denote the number of the physical and cyber nodes, respectively. $F^{P}$ and $F^{C}$ denote the number of failed physical nodes and failed cyber nodes, respectively.*

The progress of cascading failure based on load redistribution in the smart grid is as follows:

**Step 1:** A fraction 1−p of the cyber nodes experience a targeted attack and fail.

**Step 2:** The failed cyber nodes can cause the coupled physical nodes to fail due to error control commands according to the coupling relationship matrix $R_{CP}\left(i,j\right)$.

**Step 3:** The failed physical nodes can trigger load redistribution to other functioning nodes. When the load change of those physical nodes exceeds the range of their capacity, they will fail and again trigger load redistribution until the state of the power grid reaches an equilibrium.

**Step 4:** According to coupling relationship matrix $R_{PC}\left(i,j\right)$, those coupled cyber nodes also fail due to a lack of power support.

**Step 5:** The isolated nodes are removed, and the number of failed cyber and physical nodes is calculated. Finally, we obtain the survival rate *P* of the functioning nodes by calculating Formula (9).

## 5. Experiments and Analysis

### 5.1. Case Study 1: IEEE 118-Bus System

**Figure 3:**
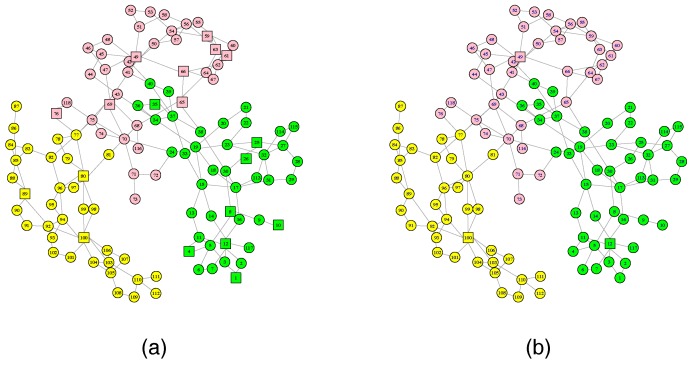
The network structure of the IEEE 118-Bus System. (**a**) The power grid. (**b**) The communication network. Different colored nodes form different subnets.

In this section, we first use the IEEE 118-Bus System to verify our approach. Figure 3a,b show a power grid and a communication network, respectively. The power grid contains 19 generators, 99 load nodes and 117 links, which is divided into three subnets. Different colored nodes form different subnets. Due to the geographic correlation between the physical nodes and the cyber nodes, we construct a communication network that contains three control centers (i.e., nodes 12, 49 and 100) and 115 sensors. The communication network also consists of three subnets, and different colored nodes form different subnets. Each control center controls its own subnet and they cooperatively control the entire power system. We assume that the coupling relationship between cyber nodes and physical nodes is the one-to-one correspondence. By changing the coupling mode between cyber and physical subnets, we are able to study the effect of different coupling modes between local coupled subnets on network robustness.

**Figure 4:**
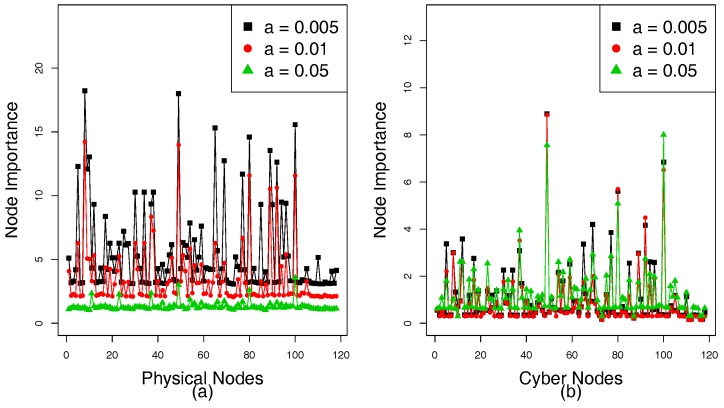
Node importance (NI) of IEEE 118-bus system according to different tolerance parameters. (**a**) NI of the physical nodes. (**b**) NI of the cyber nodes.

Figure 4a,b show the NI of the physical nodes and cyber nodes, respectively. The NI in the power grid represents the importance of a node, which relies on the size of its FNS and the DoD of coupled cyber nodes. A large NI indicates that the failed node has an important influence on the network. Tolerance α reflects the ability of a network to deals with load change caused by load redistribution. When a node’s load exceeds its capacity, it will fail. Figure 4a shows the NI of each substation that presents a downward trend as α increases. As NI of a cyber node depends on its degree and NI of the physical nodes that it controls, the NI of the cyber nodes under different α is different in Figure 4b.

The original coupling mode is a strong one-to-one coupling relationship, for instance, $p_{1}↔c_{1}$ indicates that physical node $p_{1}$ provides power supply to cyber node $c_{1}$, and $c_{1}$ also provides control support to $p_{1}$. Similarly, $p_{2}↔c_{2},\ldots ,p_{n}↔c_{n}$. In order to study the influence of different coupling modes on network robustness in detail, we divide the coupling edges into two types: the top-down and bottom-up coupling links. The top-down coupling link is monitoring/controlling edges from the cyber nodes to the physical nodes, and the bottom-up coupling link is responsible for providing power support from the physical nodes to the cyber nodes. When RCIS, ACIS, and DCIS are applied to the top-down coupling link, the bottom-up coupling link remains unchanged, for instance, $p_{1}\to c_{1},\ldots ,p_{n}\to c_{n}$, and vice versa.

**Figure 5:**
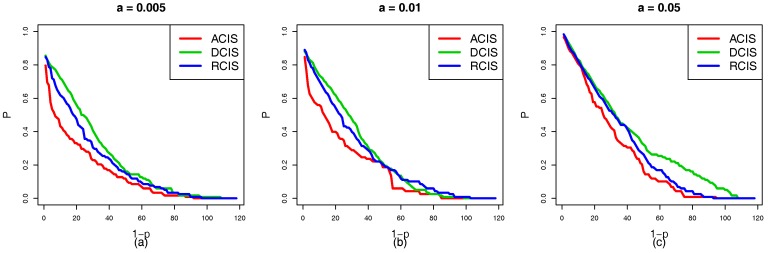
The robustness curve *P* of the smart grid according to which different coupling modes are applied to the top-down coupling link under targeted attack (**a**) Tolerance α=0.005. (**b**) Tolerance α=0.01. (**c**) Tolerance α=0.05. The red, green and blue solid curves represent ACIS, DCIS, and RCIS, respectively. The rank of robustness curves *P* is DCIS>RCIS>ACIS. This indicates that DCIS applied to the top-down coupling link is better able to enhance the robustness of the smart grid than RCIS or ACIS.

In the situation that RCIS, ACIS, and DCIS are applied to the top-down coupling link, we study the cascading failure of the smart grid with different tolerances under a targeted attack. Figure 5a–c show the robustness curve in which the red, green and blue solid lines represent the node survival rates of ACIS, DCIS, and RCIS, respectively. It is clear that the ranking of the survival rate curves *P* is DCIS>RCIS>ACIS. This means that DCIS applied to the top-down coupling link is more beneficial in enhancing the robustness of the smart grid than RCIS or ACIS. From the perspective of network science, the top-down coupling link combined with ACIS makes cyber nodes with a larger NI to couple with physical nodes with a larger NI. When these cyber nodes fail due to a targeted attack, it can lead to the failure of important physical nodes and trigger a new round of load redistribution. This causes more nodes to overload and fail. However, DCIS makes cyber nodes with a larger NI to couple with physical nodes with a smaller NI, and the failed important cyber nodes can lead to the failure of unimportant nodes that do not cause more physical nodes to fail. Therefore, DCIS applied to the top-down coupling link has a greater effect on reducing the cross-layer diffusion of cascading failures than ACIS or RCIS.

**Figure 6:**
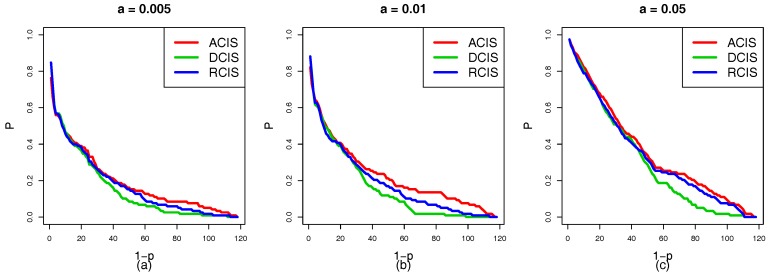
The robustness curve *P* of the smart grid according to which different coupling modes are applied to the bottom-up coupling link under a targeted attack. (**a**) Tolerance parameter α=0.005. (**b**) Tolerance parameter α=0.01. (**c**) Tolerance parameter α=0.05. The red, green, and blue solid curves represent ACIS, DCIS, and RCIS, respectively. The ranking of robustness curves *P* is ACIS>RCIS>DCIS. This indicates that ACIS applied to the bottom-up coupling link is better able to enhance the robustness of the smart grid than RCIS or DCIS.

In terms of applying RCIS, ACIS, and DCIS to the bottom-up coupling link, we analyze and research the cascading failure of the smart grid with different tolerances under a targeted attack. In Figure 6a–c, the red, green, and blue curves represent the node survival rates of ACIS, DCIS, and RCIS, respectively. It is clear that the ranking of curves *P* is ACIS>RCIS>DCIS. This means that the ACIS can effectively enhance the robustness of interdependent networks. Furthermore, ACIS can prevent the propagation of the cascading failures. From the perspective of network science, bottom-up coupling links with DCIS make physical nodes with a smaller NI to couple with cyber nodes with a larger NI, however, those insignificant physical nodes are vulnerable and easily affected by other important physical nodes. If these important nodes fail, this may lead to the failure of physical nodes with a smaller NI. Furthermore, it triggers the failure of cyber nodes with a larger NI due to a lack of power supply, and these important cyber nodes will cause more cyber nodes to fail. Meanwhile, physical nodes with a larger NI to couple with cyber nodes with a larger NI in ACIS, and these important physical nodes are highly robust and are not susceptible to failure unless they are directly attacked. Therefore, ACIS applied to a bottom-up coupling link is more beneficial in enhancing the robustness of the smart grid than RCIS or DCIS.

**Figure 7:**
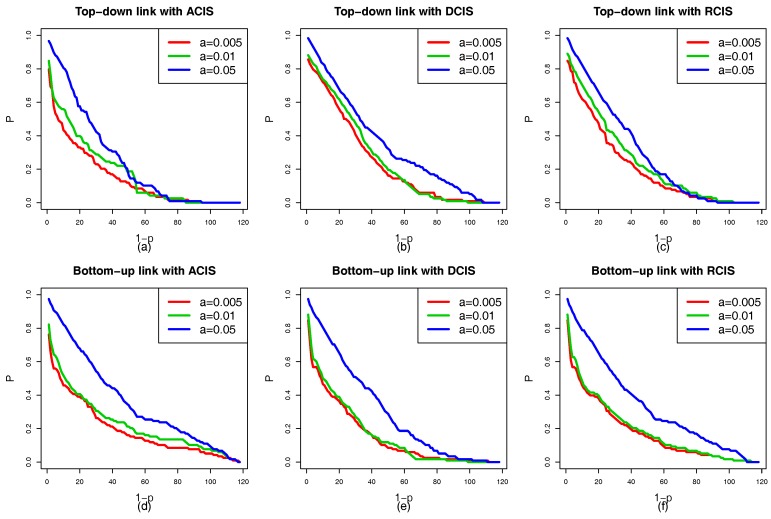
A comparison of the robustness curves *P* under different tolerances α. (**a**) Top-down coupling link with ACIS. (**b**) Top-down coupling link with DCIS. (**c**) Top-down coupling link with RCIS. (**d**) Bottom-up coupling link with ACIS. (**e**) Bottom-up coupling link with DCIS. (**f**) Bottom-up coupling link with RCIS. The red, green, and blue solid curves represent α=0.005, α=0.01 and α=0.05, respectively. The ranking of the robustness curves *P* is (α=0.05)>(α=0.01)>(α=0.005). This indicates that the tolerance α is positively related to the robustness of the smart grid.

Figure 7a–f show the situation of the cascading failure of the smart grid with the same coupling mode. It is evident that the ranking of the robustness curves *P* is α=0.05>α=0.01>α=0.005 regardless of ACIS, DCIS, or RCIS. As α increases, the capacity of interdependent networks to handle the changes of the load also increases. The tolerance α has a positive relationship with the robustness of the smart grid. This means that the high capacity benefits the robustness of the smart grid. When the tolerance α reaches 0.05, a failed physical node cannot trigger the failure of other nodes or only induces a few nodes to fail. From Figure 5, Figure 6 and Figure 7, three interesting conclusions can be drawn as follows: (I) tolerance α is positively related to the robustness of the smart grid, (II) DCIS applied to a top-down coupling link is more beneficial in enhancing the robustness of the smart grid against a targeted attack than ACIS or RCIS, and (III) ACIS applied to a bottom-up coupling link is more beneficial in enhancing the robustness of the smart grid against a targeted attack than DCIS or RCIS.

### 5.2. Case Study 2: Italian High-Voltage Electrical Transmission Network

**Figure 8:**
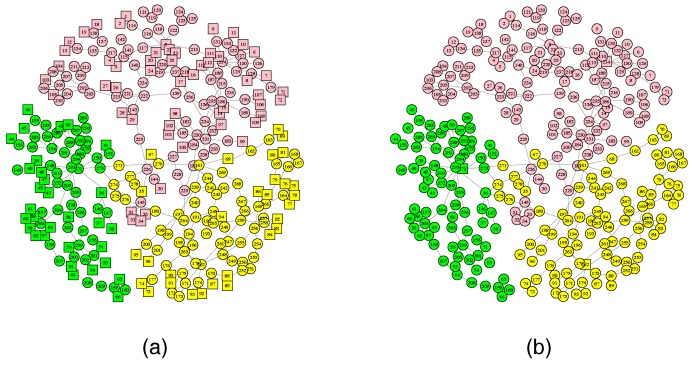
The network structure of the Italian high-voltage electrical transmission (HVIET) network. (**a**) The power grid. (**b**) The communication network. Different colored nodes form different subnets.

In order to re-verify the correctness of our conclusions, we use real network data from the Italian High-Voltage (380 kV) Electrical Transmission (HVIET) network. The network data has been taken from an analysis of the public documentation [4,53]. The HVIET network can be represented by an undirected graph of 310 substations and 361 transmission links. The topology of the HVIET network is shown in Figure 8a, where square nodes represent the generators and circular nodes represent transmission stations or distribution stations. Similarly, we construct its communication network to contain three control centers (square nodes) and 307 sensors (circular nodes), shown in Figure 8b. Different colored nodes form different subnets, and there are no coupling links between different area subnets. Therefore, our research aims to study the effect of different coupling modes applied to the dual coupling link on the robustness of interdependent networks.

**Figure 9:**
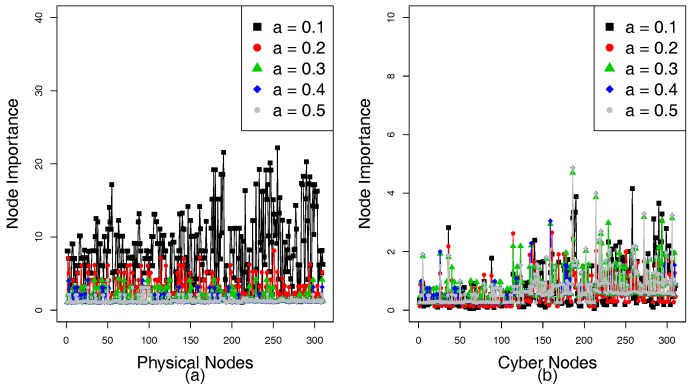
NI of the HVIET network according to different tolerance parameters. (**a**) NI of the physical nodes. (**b**) NI of the cyber nodes.

Figure 9a,b show the NI of physical nodes and cyber nodes, respectively. The NI is used to assess the impact of nodes on its network. A failed node with a larger NI may bring greater harm to the network and has a positive effect on cascading failures. Since case study 2 uses real data but case study 1 uses simulated data, this may lead to differences in tolerance between the two experiments. However, it does not affect the local coupling between the nodes in the subnets. Since the tolerance α can affect the capacity of the network to handle overloads, the NI of the physical nodes is different according to different tolerances α. Figure 9a shows that the NI is negatively correlated with α. This is mainly because as α increases, the capacity of the nodes to handle overloads also increases. A failed node cannot easily cause other nodes to fail when α reaches a certain value; therefore, the size $n(f_{i}^{P})$ of the FNS decreases. Since the NI of the physical node *i* depends on $n(f_{i}^{P})$ and the degree of degree (DoD) of coupled cyber nodes *j* and DoD of each cyber node is constant, the NI of the physical nodes decreases according to the increasing α. However, the degree of the cyber node and NI of its coupled physical node together determine its NI; therefore, there is no linear correlation between the NI of the cyber nodes and α, Figure 9b shows that NI of the cyber nodes under different tolerances is also different. When the NI of all physical and cyber nodes is obtained by Formulas (7) and (8), we can sort the importance of the physical and cyber nodes according to NI and couple the physical nodes with the cyber nodes in local subnets according to ACIS, DCIS, and RCIS.

**Figure 10:**
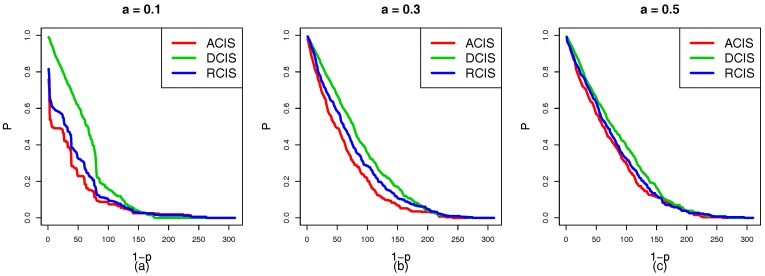
The robustness curve *P* of the HVIET network according to which different coupling modes are applied to the top-down coupling link under a targeted attack (**a**) Tolerance parameter α=0.1. (**b**) Tolerance parameter α=0.3. (**c**) Tolerance parameter α=0.5. The red, green, and blue solid lines represent ACIS, DCIS, and RCIS, respectively. The ranking of the robustness curves *P* is DCIS>RCIS>ACIS. This indicates that DCIS applied to the top-down coupling link is better able to enhance the robustness of the smart grid than RCIS or ACIS.

In order to simplify the experimental complexity and study the effect of the local coupling with ACIS, DCIS, and RCIS on the network in more detail, we only apply ACIS, DCIS, and RCIS to the top-down coupling link when the bottom-up coupling link between the cyber and physical nodes in subnets remains unchanged. Figure 10 shows the robustness curves of the HVIET network according to which different coupling modes are applied to the top-down coupling link under a targeted attack. Figure 10a–c show the situation in which the tolerance α of the HVIET network is equal to 0.1, 0.3, and 0.5, respectively. The red, green, and blue solid lines represent the robustness curves of the HVIET network with ACIS, DCIS, and RCIS, respectively. It is clear that the ranking of the robustness curves *P* is DCIS>RCIS>ACIS regardless of α=0.1, α=0.3, and α=0.5. This means that DCIS applied to the top-down coupling link is more beneficial in enhancing the robustness of the smart grid than RCIS or ACIS. That is because ACIS makes the cyber nodes with a higher NI to couple with the physical nodes with a higher NI. When those cyber nodes fail caused by a targeted attack, this will induce more cyber nodes to fail. Furthermore, important physical nodes will fail and cause more physical nodes to fail. In turn, those failed physical nodes trigger more cyber nodes to fail due to the coupling relationship. ACIS is a combination of strong physical nodes and strong cyber nodes, which will aggravate the speed of the cascading failure of interdependent networks.

**Figure 11:**
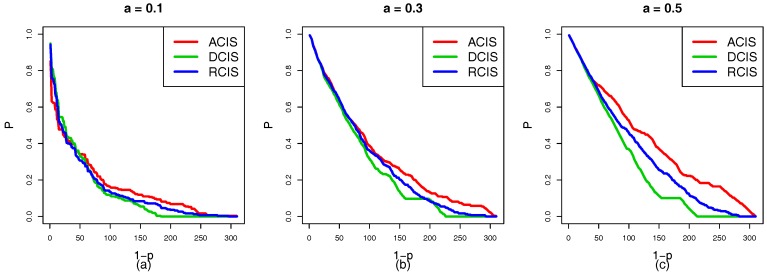
The robustness curve *P* of the HVIET network according to which different coupling modes are applied to the bottom-up coupling link under a targeted attack. (**a**) Tolerance parameter α=0.1. (**b**) Tolerance parameter α=0.3. (**c**) Tolerance parameter α=0.5. The red, green and blue solid lines represent ACIS, DCIS, and RCIS, respectively.The ranking of the robustness curves *P* is ACIS>RCIS>DCIS. This indicates that ACIS applied to the bottom-up coupling link is better able to enhance the robustness of the smart grid than RCIS or DCIS.

Similarly, we only apply different coupling modes to the bottom-up coupling link, while the top-down coupling link remains unchanged. Figure 11 shows the robustness curves of the HVIET network according to which the different coupling modes are applied to the bottom-up coupling link under a targeted attack. Figure 11a–c show the situation in which the tolerances α of the HVIET network are equal to 0.1, 0.3, and 0.5, respectively. The red, green, and blue solid lines represent the robustness curves *P* of the HVIET network with ACIS, DCIS, and RCIS, respectively. However, we find a counterintuitive conclusion that ACIS applied to the bottom-up coupling link is better able to enhance the robustness of the smart grid than DCIS or RCIS. It is evident that the ranking of the robustness curves *P* is ACIS>RCIS>DCIS regardless of α=0.1,α=0.3, and α=0.5. This is because any failed physical nodes may cause the physical nodes with a smaller NI to fail, which further leads to the failure of the cyber nodes with a larger NI. If the important physical nodes are coupled with the important cyber nodes, those cyber nodes fail only when the important physical nodes fail.

**Figure 12:**
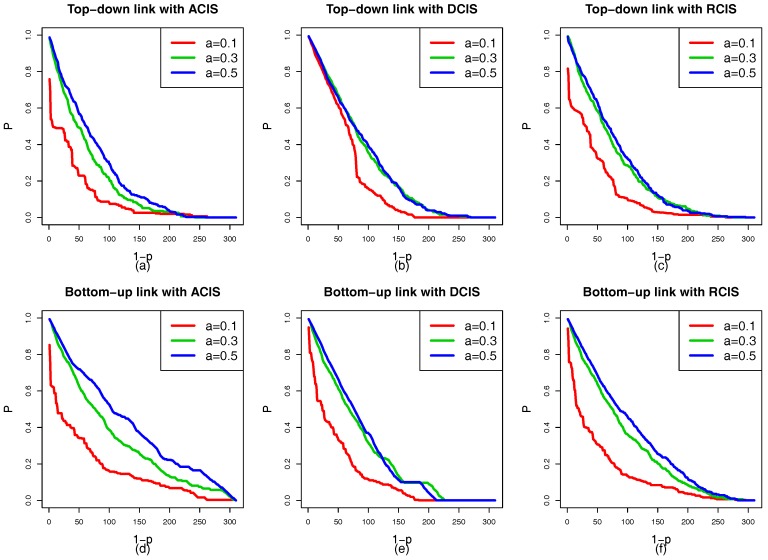
A comparison of robustness curves *P* of the HVIET network under different tolerance α. (**a**) Top-down coupling link with ACIS. (**b**) Top-down coupling line with DCIS. (**c**) Top-down coupling link with RCIS. (**d**) Bottom-up coupling link with ACIS. (**e**) Bottom-up coupling link with DCIS. (**f**) Bottom-up coupling link with RCIS. The red, green, and blue solid lines represent the robustness curves under α=0.1, α=0.3, and α=0.5, respectively. The ranking of robustness curves *P* is (α=0.5)>(α=0.3)>(α=0.1). This indicates that the tolerance α is positively related to the robustness of the smart grid.

In addition to the coupling mode, the tolerance α is an important factor which affects the robustness of interdependent networks. Figure 12a–f show that the ranking of the robustness curves *P* is (α=0.5)>(α=0.3)>(α=0.1) regardless of the coupling links (top-down and bottom-up) and coupling modes (ACIS, DCIS, and RCIS). This means that a bigger α is also better able to enhance the robustness of interdependent networks. The same conclusions are obtained from the case studies 1 and 2, which can be summarized as follows: (I) DCIS applied to the top-down coupling link is more beneficial in enhancing the robustness of the smart grid against a targeted attack than RCIS or ACIS, (II) ACIS applied to the bottom-up coupling link is better able to enhance the robustness of the smart grid against a targeted attack than RCIS or DCIS, and (III) the robustness of the smart grid can be improved by increasing the tolerance α against a targeted attack.

## 6. Conclusions

This paper has proposed a strategy that combines different coupling modes with dual coupling links in order to increase the robustness of smart grid. Load redistribution, local coupling in subnets, different coupling modes, and dual coupling link have fully been considered in an improved failure model. NI was used to assess the impact of nodes on its single network and is used as an evaluation index to connect cyber node to physical node in order to generate assortative, disassortative, and random couplings. There are two types of dual coupling links: the top-down coupling link (C→P) and the bottom-up coupling link (P→C). ACIS, DCIS, and RCIS were applied to the top-down coupling link and bottom-up coupling link for studying the robustness of interdependent networks. In addition, we proposed a reasonable local coupling mechanism according to which the cyber and physical networks are divided into small subnets, and the cyber nodes are only allowed to be coupled with the physical nodes in the same geographical area. This avoids the high cost and irrationality of global coupling between cyber and physical nodes due to long-distance. We examined two case studies to research the effect of different coupling modes on the robustness of interdependent networks and have got the same conclusions that a high tolerance α, a top-down coupling link with DCIS, and a bottom-up coupling link with ACIS can enhance the robustness of the smart grid.

Previously, many literatures have studied the failure mechanisms of symmetry networks and few studies have been done on the cascading failure of asymmetric networks. In fact, there is an overload failure problem in the communication network. When the data flow of a node exceeds its processing capacity, it will refuse to provide the service and become invalid. Therefore, the load redistribution of cyber nodes should also be fully taken into account in the failure model of interdependent networks. This means that the data flow load that a failed cyber node is responsible for forwarding will be distributed to its neighbor nodes. In addition, the influence of global coupling and local coupling on interdependent networks is a very interesting research direction and different network types have a significant impact on the robustness of interdependent networks. As such, the more complicated models should be established to study the effect of different coupling modes, dual coupling link, coupling strength, load redistribution of cyber and physical nodes, and asymmetric coupling between the cyber and physical networks on the robustness of interdependent networks.

In the future, coordinated cyber-physical attack, dynamic cross-layer attack path identification, a coordinated detection mechanism, and a network attack and defense confrontation on infrastructure will be the areas of interest. In fact, the main factor affecting the safety of infrastructure is human input; therefore, the three-layer social-cyber-physical coupling relationship should also be examined to establish a framework to protect critical infrastructures.

## Abbreviations

The following abbreviations are used in this manuscript:

ACIS	Assortative Coupling in Subnets
DCIS	Disassortative Coupling in Subnets
RCIS	Random Coupling in Subnets
NI	Node Importance
FNS	Failure Node Set
EMS	Energy Management Systems
SCADA	Supervisory Control and Data Acquisition
